# Healthcare professionals' perceptions of system preparedness during public health emergencies: a path analysis of mental health impacts

**DOI:** 10.3389/fpubh.2025.1449207

**Published:** 2025-04-17

**Authors:** Runze Huang, Xueting Ding, Anlong Li, Guodong Nie, Ling Cheng, Yajing Li, Wei Gao, Han Ge, Mingjun Zhang, Huaidong Cheng

**Affiliations:** ^1^Department of Oncology, The Second Hospital of Anhui Medical University, Hefei, Anhui, China; ^2^Second School of Clinical Medicine, Anhui Medical University, Hefei, Anhui, China; ^3^Department of Health, Society, and Behavior, Joe C. Wen School of Population and Public Health, Susan and Henry Samueli College of Health Sciences, University of California, Irvine, Irvine, CA, United States; ^4^Medical Intensive Care Unit, The First Affiliated Hospital of Anhui University of Chinese Medicine, Hefei, Anhui, China; ^5^Department of Respiratory, The First Hospital of Anhui Medical University, Hefei, Anhui, China; ^6^Department of Medicine, Dingyuan County General Hospital, Chuzhou, China; ^7^School of Nursing, Anhui Medical University, Hefei, Anhui, China; ^8^The Third School of Clinical Medicine, Southern Medical University, Guangzhou, China; ^9^Department of Oncology, Shenzhen Hospital of Southern Medical University, Shenzhen, Guangdong, China

**Keywords:** perceived inadequate system preparedness, stress, resilience, negative emotions, healthcare professionals, chain-mediation analysis, easing policy, COVID-19

## Abstract

**Background:**

The easing of COVID-19 policies in China appears to have been inadequately prepared, leading to a profound shift in the mental wellbeing of healthcare professionals following the lifting of these measures. Our study aims to investigate the pathways underlying negative emotions experienced by healthcare professionals due to perceived inadequate system preparedness, aiming to enhance their mental health protection and facilitate more effective responses during future large-scale public health crises.

**Methods:**

A total of 826 healthcare professionals were enrolled. Depression symptoms, anxiety symptoms, perceived stress, resilience, perceived inadequate system preparedness were measured in our research.

**Results:**

The prevalence of depression and anxiety symptoms among healthcare professionals were 32.1 and 16.2%, respectively, during the concentrated outbreak of COVID-19 in China after easing policy. The chain mediation analysis reveals that perceived inadequate system preparedness significantly directly predicts depression or anxiety symptoms among healthcare professionals, indirectly through the mediating role of stress, as well as via the chain mediation of stress and resilience. However, it does not predict these symptoms through the mediator of resilience alone. Furthermore, contracting COVID-19 directly predicts depression symptoms.

**Conclusions:**

Perceived inadequate system preparedness can have a detrimental impact on negative emotions through various channels. When facing the potential outbreak of a large-scale public health event in the future, it is crucial to implement measures such as providing psychological counseling, increasing risk allowances, and ensuring an adequate supply of personal protective equipment to be better prepared. Additionally, psychosocial interventions should be implemented to enhance the resilience of healthcare professionals and safeguard the mental wellbeing of those infected with COVID-19, etc.

## 1 Introduction

It has been nearly 4 years since COVID-19 was declared a pandemic by the World Health Organization (WHO) on 11 March 2020 (1). In the past 4 years, the COVID-19 pandemic has brought illness, death and enormous mental stress to people. As of March 31, 2024, over 774 million confirmed cases and over 7 million deaths have been reported globally (2). In addition to the toll on human lives, economic downturns (3), and trade disruptions (4), the COVID-19 epidemic has also inflicted significant psychological trauma on individuals (5), particularly healthcare professionals who courageously serve at the forefront (6). Although the current epidemic situation remains generally stable, sporadic outbreaks persist in certain regions. Furthermore, a significant proportion of individuals continue to endure the enduring physical and psychological repercussions of “Long-COVID-19” (7). It can be asserted that, whether during the course of the epidemic or in its aftermath, the prevailing pathogen or its variants exert an immense and indelible impact on the entire populace, especially during an intensified outbreak period (8). The impact and psychological trauma experienced by medical staff are even more pronounced (9). They may exhibit symptoms indicative of depressive disorders, anxiety disorders, occupational burnout, or even post-traumatic stress disorder (PTSD) (10). Thus, drawing from past experiences, especially the period of concentrated outbreak, is imperative to mitigate losses and trauma stemming from future outbreaks of large-scale infectious diseases. And it is of utmost importance to prioritize the safeguarding of healthcare professionals' mental wellbeing, as they serve as the frontline “firefighters” whose compromised state would jeopardize our health.

During the initial phase of the COVID-19 outbreak, the simultaneous increase in infected individuals and inadequate implementation of certain protective measures exacerbated psychological challenges among healthcare professionals. In the initial phase of the Wuhan outbreak, Kang et al. (11) identified a significant impact on the mental health of healthcare professionals due to exposure to infection and inadequate provision of mental health education, with ~28.6% of medical workers experiencing moderate-to-severe mental health disorders. Subsequently, as the virus rapidly disseminated, COVID-19 outbreaks ensued in diverse global locations. A study in Poland, Europe found that during a surge in infected patients, the provision of personal protective equipment by employers can directly predict job burnout, emotional exhaustion and job satisfaction of health care workers, with 24.95 and 16.5% of them having anxiety and depression, respectively (12). While a study in Iran, the Middle East found 44.8%, 43%, and 34.8% of healthcare workers having depression, anxiety and stress symptoms during the peak of the epidemic, respectively (13). After the epidemic reached its peak, a period of stability ensued, characterized by a gradual decline in the number of infected individuals and effective implementation of protective measures, especially in China (14). Consequently, there has been some alleviation of negative emotions among medical personnel. During the regular epidemic control stage, a study revealed that only 5.5% experienced symptoms indicative of moderate to severe anxiety (15). After the onset of 2022, the highly transmissible Omicron variant rapidly disseminated, while concurrently exhibiting a gradual attenuation in its pathogenicity (16). Therefore, a novel transformation has ensued within the realm of the COVID-19 pandemic.

Due to the changes in the COVID-19 epidemic, the Chinese government announced 20 measures on November 11, 2022. On December 7, the “10 new measures” were announced (17). These measures marked a significant shift from China's strict “zero-COVID” policy. The 20 measures included reducing quarantine periods and limiting mass testing, while the 10 new measures further relaxed restrictions by allowing home isolation for mild cases and reducing PCR testing requirements. This study's timing after these policy changes was deliberately chosen to examine the healthcare system's response to the subsequent surge in cases, providing unique insights into system preparedness, especially related with the perceptions of health professionals, that weren't visible during the controlled outbreak phase. However, certain policy preparation measures appear to be insufficient, such as the inadequate availability of medical resources like masks and drugs, the overwhelming influx of COVID-19 patients within a short timeframe at medical facilities, and the absence of psychological interventions. After the abrupt lifting of lockdown and other restrictive measures, there was a sharp increase in infection cases and extensive dissemination of the virus nationwide (18). This has imposed a significant burden on healthcare institutions within a compressed timeframe, resulting in numerous healthcare professionals experiencing substantial psychological strain. According to a network analysis, the prevalence of depression and anxiety among healthcare workers increased from 35.32 and 48.02%, respectively, during the first wave of the COVID-19 pandemic following the relaxation of control measures, to 71.74 and 72.75% during the second wave (19). It is evident that following the easing policy of China, there was a higher prevalence (particularly the second peak) of negative emotional symptoms among medical personnel compared to their counterparts in the aforementioned representative cities or countries during their respective peak periods of the epidemic. Additionally, Blasi et al. discovered that the psychological states of Italian populations remained stable during both the lockdown period and post-lockdown phase (20). In contrast, Chinese medical personnel experienced a significant shift in their psychological wellbeing following the relaxation of restrictions. Compared to the stringent control measures implemented prior to epidemic prevention and control, negative emotions among Chinese medical personnel exhibited a sharp increase, likely attributed to inadequate preparations for policy relaxation. Therefore, it is imperative to draw lessons from China's experience with policy relaxation in order to enhance mental health support for medical personnel during future large-scale epidemics.

These insufficient preparations can be attributed to inadequate system preparedness and related perceptions during the pandemic. Although inadequate system preparedness lacks a precise definition, it might be categorized into the subsequent dimensions: (1) Insufficient provision of personal protective equipment (PPE) (21); (2) Insufficient surge capacity in hospitals, characterized by an overwhelming number of patients and inadequate availability of wards and beds (22); (3) Excessive workload and prolonged working hours are prevalent issues faced by medical personnel (23); (4) Comprehensive psychological health interventions are imperative for healthcare professional, encompassing pre-employment training, formulation of protocols for the utilization and management of protective equipment, rational organization of recreational activities, and establishment of dedicated rest areas (24); (5) Others: healthcare professionals necessitate hazard pay (25); mitigate nosocomial transmission and cross-contamination of the virus (23, 26); fortify hand hygiene practices (27) etc. In this study, we have developed the “perceived inadequate system preparedness” questionnaire, reflecting the most critical and urgent concerns of healthcare professionals, to assess the lack of preparedness during an epidemic outbreak after easing policy of China. While healthcare system preparedness encompasses multiple dimensions including organizational structures, risk assessment systems, and community engagement as outlined by WHO guidelines (28), this study specifically focuses on healthcare professionals' perceptions of system preparedness through their direct experiences. This perspective provides valuable insights into how frontline workers experience and evaluate their healthcare system's readiness during crisis.

These factors of unpreparedness can give rise to a multitude of adverse emotional symptoms among healthcare professionals. For instance, two studies conducted in Ghana have demonstrated that the perceived lack of preparedness among healthcare professionals can significantly diminish their job satisfaction, leading to elevated levels of stress and burnout (29, 30). Although limited in scope, existing studies have consistently indicated a correlation between perceived inadequate preparedness and the manifestation of negative emotions during epidemic situations. Insufficient provision of medical protective equipment, limitations on contact with family members, transfers to different wards, and excessive workload can all contribute to the development of mental health issues (depression, anxiety, insomnia, etc.) among healthcare professionals, as indicated by a systematic review (31). Conversely, a nationwide survey revealed that the presence of effective prevention and control measures as well as efficient health systems served as protective factors against psychological distress (32). However, the existing literature has predominantly focused on individual exploration of which measure of preparedness predicts negative emotions among healthcare professionals. In contrast, the two studies conducted in Ghana that were previously mentioned did not investigate the pathways through which total preparedness affects negative emotions, particularly without establishing a classic mediation model. Therefore, our objective was to establish a mediating model in order to investigate the pathway of the impact of overall perceived inadequate system preparedness on negative emotions. And given that depression and anxiety are the prevailing negative emotional symptoms, we have designated them as dependent variables in the model, with perceived inadequate system preparedness as independent variable. In conclusion, our first hypothesis is:

Hypothesis 1: Perceived inadequate system preparedness can directly impact the depression and anxiety symptoms among healthcare professionals.

The elucidation of the impact of perceived inadequate system preparedness on healthcare professionals' negative emotions remain elusive. However, insufficient preparation can significantly elevate the stress levels experienced by healthcare professionals. According to Azizi et al. (13), the number of daily working hours has the potential to serve as a predictor for stress levels. Additionally, Afulani et al. (29) discovered a positive correlation between healthcare workers' perceived readiness and job satisfaction, while also identifying a negative correlation between job satisfaction and stress levels. Furthermore, extensive evidence has consistently demonstrated a strong correlation between stress levels and the experience of negative emotions. Experiencing stressful or traumatic events can precipitate the onset of depressive and anxiety disorders (33, 34). Specifically, the COVID-19 pandemic represents a source of traumatic stress that may engender symptoms of anxiety, depression, post-traumatic stress disorder (PTSD), and burnout among healthcare professionals (35). Therefore, our second hypothesis is:

Hypothesis 2: Perceived inadequate system preparedness can exert an impact on depression and anxiety symptoms among healthcare professionals by means of stress mediation.

In contrast to stress and perceived inadequate preparedness, resilience serves as a protective factor against negative emotions of healthcare professional (36). Although the relationship between resilience and perceived inadequate system preparedness has not been investigated, extensive research has unequivocally established the association between resilience and negative emotions. The resilience construct exhibits a significant negative correlation with adverse affective states, such as depression and anxiety (37), while being underpinned by neural cognitive mechanisms involved in the regulation of emotions (38). Lin et al. (39) discovered that the resilience exhibited by medical personnel from outside Wuhan who provided assistance during the COVID-19 outbreak served as a protective factor against anxiety and depression. Based on the above hypothesis, perceived inadequate system preparedness could significantly impact the negative emotions experienced by healthcare professionals. Moreover, it is evident that resilience, which exhibits a strong association with negative emotions, may act as a mediating factor in this relationship. Therefore, we propose our third hypothesis:

Hypothesis 3: Perceived inadequate system preparedness can exert an impact on depression and anxiety symptoms among healthcare professionals by influencing resilience as a mediating factor.

There also exists a significant association between stress and resilience among healthcare professionals.

Barzilay et al. (40) revealed a negative correlation between higher levels of resilience and the experience of elevated stress or worry in relation to COVID-19. Furthermore, based on Hypothesis 3, it is evident that resilience exhibits a strong association with negative emotions. Chen et al. (41) have provided empirical evidence supporting the mediating role of resilience in the relationship between perceived stress and adverse emotional states such as depression and anxiety. By integrating the theoretical perspective of Hypothesis 2 with these aforementioned findings, we propose Hypothesis 4:

Hypothesis 4: Stress and resilience have a chain-mediating effect between perceived inadequate system preparedness and depression or anxiety symptoms among healthcare professionals.

**Figure 1:**
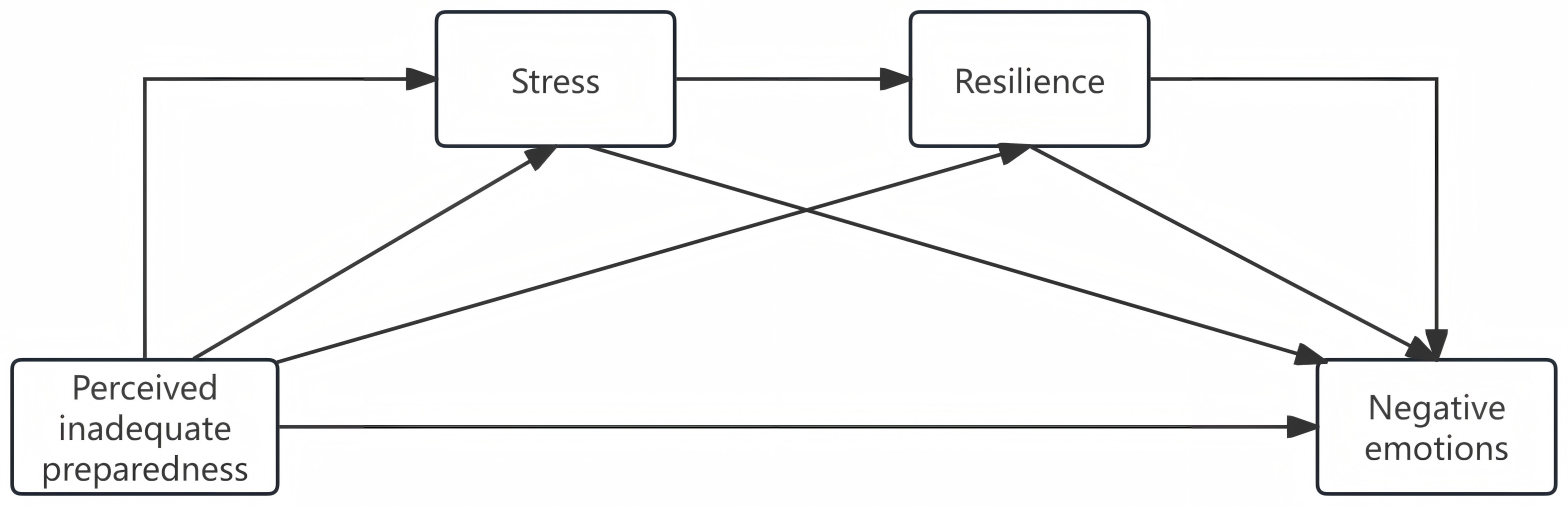
Hypothesized model.

In summary, the objective of this study is to investigate the impact of perceived inadequate system preparedness during the peak period of an epidemic on healthcare professionals' negative emotions, stress levels, and resilience, as well as to examine whether stress and resilience can act as mediators in this process. In this study, healthcare professionals include doctors, nurses, medical fellows in training, and other allied health support staff. This comprehensive definition ensures we capture the full spectrum of professionals involved in healthcare delivery during the pandemic. The aim of this study is to contribute valuable insights for future mass epidemic responses and enhance the mental wellbeing of healthcare professionals. Based on the aforementioned four hypotheses, we have established a chain mediator model (Figure 1). To the best of our knowledge, the present study represents the pioneering attempt in investigating this field.

## 2 Methods

### 2.1 Study design

The survey was based on non-probability sampling design and was conducted from December 22, 2022 to January 20, 2023. Online questionnaires (selected questions in Chinese version and English version are in Supplementary material 1) were administered on the online survey platform (https://www.wjx.cn/) and sent to large hospitals and primary hospitals in seven provinces. We surveyed healthcare professionals after the easing policy was implemented. Healthcare professionals mainly include doctors, nurses, fellows in training (including medical school graduates awaiting residency placement, medical students completing required clinical rotations, and residents) and others (other allied health professionals including pharmacists, technicians, and other clinical support personnel). During the survey, a quick response code (QR code) linking to the online questionnaire was sent to the WeChat working group of the above hospitals.

To ensure the accuracy and validity of the data, we set up intelligent logical checks in a computer backend system to identify and reject invalid questionnaires that were (1) completed < 2 min, indicating insufficient attention to questions, or (2) with only identical responses to all questions, indicating pattern responding. Each person could only complete the questionnaire once.

### 2.2 Measurements

The questionnaire included the following six sections, Sociodemographic data, Patient Health Questionnaire-9, Generalized Anxiety Disorder 7-Item Scale, Perceived Stress Scale-10, Brief Resilient Coping Scale and Perceived Inadequate System Preparedness. When developing the “Perceived Inadequate System Preparedness” scale, we specifically selected items that healthcare professionals are most concerned about and directly associated with inadequate preparedness and related perceptions, then aggregated their scores to obtain a total score. Higher scores on this scale indicate a greater absence of perceived preparation measures.

#### 2.2.1 Sociodemographic data

Sociodemographic data included gender, age, type of work, work experience, education, current situation, COVID-19 infection status, marriage, monthly income. Current situation refers to whether work in hospital, including working, staying at home due to the pandemic, and being in quarantine in a hospital or elsewhere.

#### 2.2.2 Patient health questionnaire-9 (PHQ-9)

PHQ-9 was the most widely used instrument for screening depression and depression severity in primary health care (42). Each of the 9 items was divided into four-point degrees of the scale (0 = not at all; 1 = some of the time; 2 = more than half the time; 3 = nearly every day) in the past 2 weeks. The total score ranged from 0 to 27. A cut-off value of 10 points has high sensitivity and specificity (43). Therefore, 10 points were taken as the cut-off point in this study. A score of ≥10 is considered moderate-to-severe depression symptoms or depressive disorder.

#### 2.2.3 Generalized anxiety disorder 7-item (GAD-7) scale

GAD-7 is an effective tool for screening generalized anxiety disorder in clinical studies with good reliability and validity (44). Each of the 7 items was rated on four-point degrees of the scale (0 = not at all; 1 = some of the time; 2 = more than half the time; 3 = nearly every day) in relation to the past 2 weeks. The severity score ranged from 0 to 21. It was of more clinical significance to use 10 as the cut-off points for anxiety symptoms (45). As a result, 10 points were taken as the cut-off point in this study. A score of ≥10 is considered moderate-to-severe anxiety symptoms or anxiety disorder.

#### 2.2.4 Perceived stress scale-10 (PSS-10)

The Perceived Stress Scale was one of the most popular tools for measuring mental stress (46). A variety of studies have used the PSS to measure COVID-19 stress (47–49). It had three versions. In those, the psychometric properties of PSS-10 was superior to PSS-14 and PSS-4 (46). The PSS-10 consisted of six negative items and four positive items. Each item was divided into a five-point frequency scale (0 = never; 1 = almost never; 2 = sometimes; 3 = fairly often; 4 = very often) in relation to the past month. PSS scores are obtained by reversing the scores on the four positive items, 0 = 4, 1 = 3, 2 = 2, etc., and then summing across all 10 items, ranging from 0 to 40 (50). Higher scores showed a higher level of perceived stress. As there is a lack of studies proposing a standard cut-off score to grade stress (49). We categorized the PSS scores into four quartiles as four levels. The lower quartile (the first level) includes scores ≤ 13, the second quartile (the second level) ranges from 14 to 17, the third quartile (the third level) ranges from 18 to 20, and the upper quartile (the fourth level) includes scores that are >20 out of a possible 40.

#### 2.2.5 Brief resilient coping scale (BRCS)

The Brief Resilient Coping Scale (BRCS) was originally designed to assess resilient coping, with sufficient reliability (51). Its psychometric properties are validated in multiple countries (52–54). It has 4 items, using a 5-point Likert scale “from ‘1' = describes me not at all to ‘5' = describes me very well” (51). The total score ranges from 4 to 20; the higher the score, the more resilience (55). Scores of the BRCS was divided into tertiles as low (4–13), moderate (14–16), and high (17–20), consistent with the original study and other studies (47, 51, 56, 57).

#### 2.2.6 Perceived inadequate system preparedness

A Likert-type scale was developed based on the extensive literature review and a comprehensive analysis of the most critical and pressing issues currently faced by healthcare professionals. The question is “Which of the following things do you think your bad mood comes from and give a rating (The higher the score, the more you worried and more in line with the current situation)”. There were five items in total, including “Lack of psychological counseling measures”, “Lack of masks, medicine and other supplies”, “Salaries need to be improved during the epidemic”, “An excess of infected patients”, and “Working long hours with no breaks”. The score for each option was set to 0–5. Each item in the scale was scored separately. The scores for all items are added together as a total score. This scale was developed to assess healthcare professionals' perceptions of their healthcare system's preparedness during the COVID-19 response. While recognizing that system preparedness encompasses broader organizational and structural elements as outlined by WHO (28), this questionnaire focuses on frontline workers' experiences of key preparedness indicators.

Cronbach's alpha was used to measure internal consistency and explorative factor analysis was applied to test the factor structure within the questionnaire. The Cronbach's alpha coefficient of the questionnaire was 0.916. Revisions were not made to any of the items as they did not increase the value of Cronbach's alpha if they were deleted, thus warranting their inclusion in the questionnaire (Supplementary Table S1). The value of Kaiser–Meyer–Olkin (KMO) Measure of Sampling Adequacy of the questionnaire was 0.886. In the Bartlett's test of sphericity, the approximate Chi-square value was 3,038.803 (*p* < 0.001). These KMO and Bartlett's test indicated that 5 items of Perceived inadequate system preparedness were eligible for factor analysis. The principal component factor analysis reveals that the five items are associated with a common dimension (namely perceived inadequate system preparedness), which accounts for 75.17% of the total variance. Moreover, all five items exhibit factor loadings exceeding 0.7 and coefficient of communalities surpassing 0.5. As there is only one dimension, rotational adjustments are unnecessary. The results of the exploratory factor analysis are presented in Supplementary Table S2. In conclusion, the 5 items in this questionnaire demonstrate robust internal consistency and construct validity, thus affirming their reliability and soundness.

### 2.3 Statistical analysis

**Figure 2:**
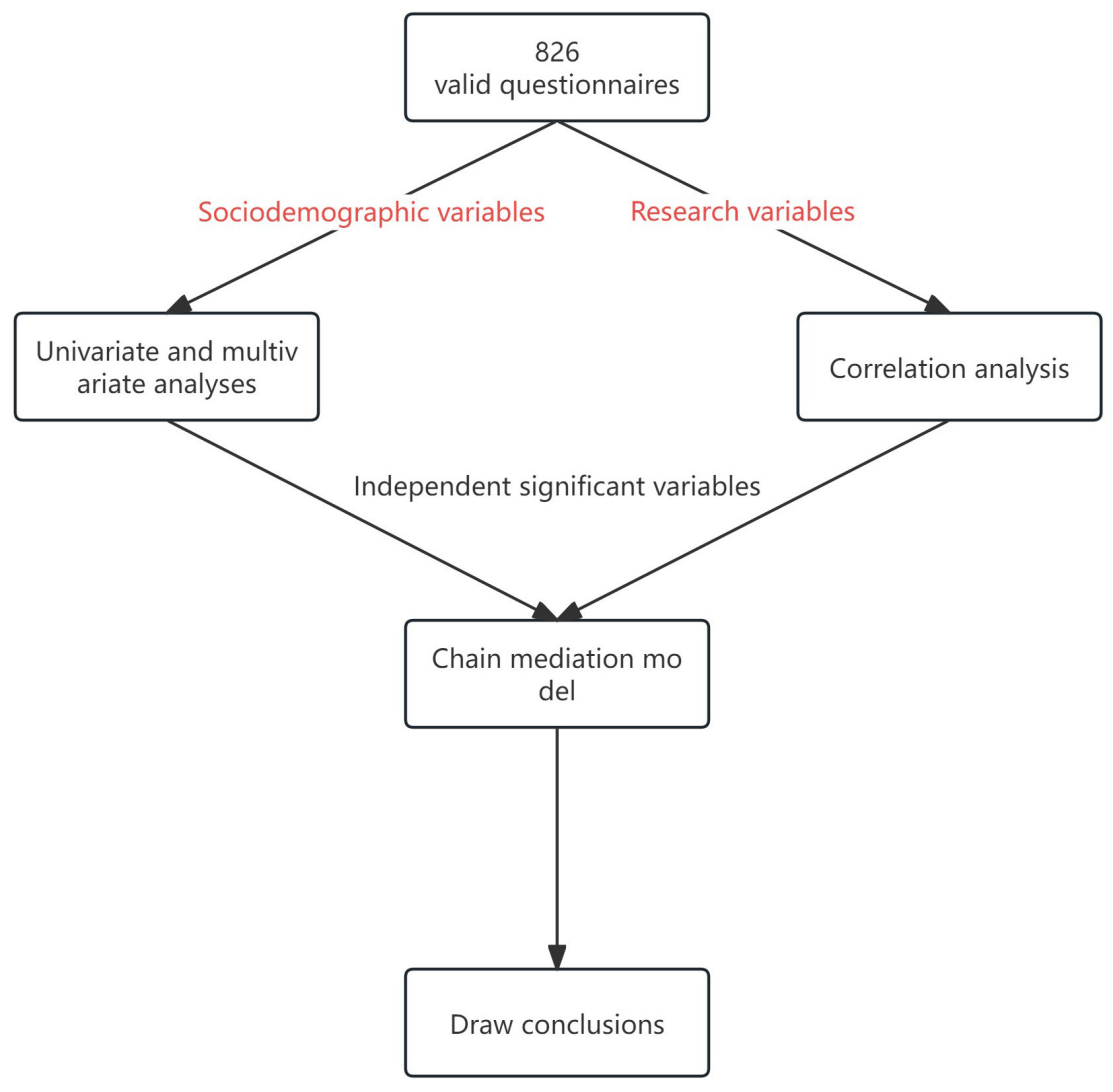
Design of the research route.

The diagram illustrating the design of the research route is presented in Figure 2. Descriptive statistics were used to summarize the sample in terms of sociodemographic information, express with corresponding numbers and percentages.

The sociodemographic variables were subjected to univariate and multivariate analyses. Univariate analysis methods included chi-square test and rank-sum test. Chi-square tests are used for binary or unordered multiclass variables. Rank sum test is used for those rank variables. The statistically significant variables were included in multivariate analysis. A stepwise logistic regression model is employed in multivariate analysis to identify independent factors associated with symptoms of depression or anxiety. Chi-square and rank-sum tests report chi-square values and corresponding *p*-values, and logistic stepwise regression reports adjusted OR values, 95% confidence intervals, and *p*-values.

The chain mediator model was constructed using Model 6 in the SPSS macro program to empirically examine the four hypotheses posited in this study. Before building the model, the research variables undergo correlation analysis to determine their support for the establishment of this model, and sociodemographic variables that were independently associated with negative emotions in the stepwise logistic regression will be included as control variables in the model for adjustment. During the process of model construction, the Bootstrap method was used to test for significance of the regression coefficient, and the confidence interval corrected for robust standard error and 95% bias was obtained, if the confidence interval did not contain zero, the significance test *p* < 0.05 indicated that the effect was statistically significant.

While a priori sample size calculation was not conducted due to the novel nature of our mediation model, *post-hoc* power analyses were performed (58). For the depression symptoms model, power analyses yielded 1.00 for the stress mediation path (perceived system preparedness-stress-depression symptoms) and 0.97 for the stress-resilience chain mediation path (perceived system preparedness-stress-depression symptoms). For the anxiety symptoms model, power analyses showed 1.00 for the stress mediation path and 0.81 for the stress-resilience chain mediation path, indicating adequate statistical power for detecting the hypothesized mediation effects.

All statistical tests were two-sided and *p* < 0.05 was considered significant. The data was analyzed by SPSS 22.0 (IBM Corp. New York, USA.). In addition, we used Harman's one factor test to check for common method bias in this study. The results showed that the first factor accounted for 33.601% of the total variance (< 40% threshold value). Therefore, no significant common method bias exists in this study.

## 3 Results

A total of 1,056 questionnaires were collected. Questionnaires were excluded (230) if all responses were selected from the same option and if the duration of completion was exceptionally brief, a total of 826 valid questionnaires were collected.

### 3.1 Descriptive statistics of sociodemographic information

Of the respondents, 66.3% were female and 33.7% were male. Most of them were 18–25 years old (39.2%) and 31–40 years old (23.2%). There were 36.0% fellows in training, 36.4% doctors, 16.1% nurses and 11.5% other healthcare professions. Their work experience ranged from just after entry (< 2 years) to more than 10 years. 59.4% had bachelor as their highest degree, and 90.3% were infected with COVID-19. The majority monthly income was below 6,000 yuan (66.8%) (Table 1).

**Table 1:** Demographic characteristics of the respondents (*N* = 826).

**Demographics**	
**Sex**	
Male	278 (33.7%)
Female	548 (66.3%)
**Age, years**	
18–25	324 (39.2%)
26–30	133 (16.1%)
31–40	192 (23.2%)
41–50	98 (11.9%)
>50	79 (9.6%)
**Type of work**	
Fellows in training	297 (36.0%)
Doctors	301 (36.4%)
Nursers	133 (16.1%)
Others^a^	95 (11.5%)
**Work experience, years**	
< 2	327 (39.6%)
2–5	106 (12.8%)
6–10	92 (11.1%)
>10	301 (36.4%)
**Education**	
Junior college	213 (25.8%)
College	491 (59.4%)
Master and above	122 (14.8%)
**COVID-19 infection status**	
Uninfected	80 (9.7%)
Infected with obvious symptoms	265 (32.1%)
Almost or fully recovered	481 (58.2%)
**Marriage**	
Unmarried/divorced	405 (49.0%)
Married	421 (51.0%)
**Monthly income, yuan**	
< 6,000	552 (66.8%)
6,000–10,000	217 (26.3%)
>10,000	57 (6.9%)

^a^Members of the support staff, including personnel in administrative, medical, and pharmacy departments, among others.

### 3.2 Univariate analysis and logistic regression analysis of sociodemographic variables

#### 3.2.1 Factors associated with depression symptoms

A total of 265 (32.1%) participants reported moderate-severe depression symptoms. Univariate analysis showed that type of work, gender, age, education, COVID-19 infection status, and marital status were statistically associated with depression symptoms (*p* < 0.05).

The above variables were included in the stepwise logistic regression model. It showed that compared with uninfected with COVID-19, infected with symptoms (OR = 4.61, 95%CI: 1.95–10.91, *p* < 0.001), COVID-19 almost or fully recovered (OR = 3.62, 95%CI: 1.56–8.41, *p* = 0.003) were more likely to report depression symptoms. Besides, compared to male, female (OR = 1.48, 95%CI: 0.99–2.21, *p* = 0.053) appeared to more likely have depression symptoms (close to α = 0.05 significance level; Supplementary Table S3).

#### 3.2.2 Factors associated with anxiety symptoms

A total of 134 (16.2%) participants reported moderate-severe anxiety symptoms. Univariate analysis showed that type of work, age, work experience, marital status, and monthly income were statistically associated with anxiety symptoms (*p* < 0.05).

The above variables were included in the stepwise logistic regression model. It showed that compared with 18–25 years old, 26–30 years old (OR = 3.19, 95%CI: 1.41–7.24), 31–40 years old (OR = 6, 95%CI: 2.1–17.12), 41–50 years old (OR = 4.98, 95%CI: 1.55–15.98), and >50 years old (OR = 6.23, 95%CI: 1.69–22.92) were more likely to report anxiety symptoms (*p*-value all < 0.01). Among them, aged 31–40 and over 50 seemed to be at greatest risk. Besides, compared to single, married (OR = 0.35, 95%CI: 0.14–0.87, *p* = 0.024) appeared to be a protective factor (Supplementary Table S4).

### 3.3 Correlation analyses of research variables

Supplementary Table S5 presents the descriptive statistics (i.e., means and standard deviations) for each research variable, along with the correlation matrix among these variables. Results indicated that perceived inadequate system preparedness was positively correlated with stress (*r* = 0.494), depression symptoms (*r* = 0.761), and anxiety symptoms (*r* = 0.657). Stress also positively correlated with depression (*r* = 0.620) and anxiety symptoms (*r* = 0.662). While resilience was negatively correlated with perceived inadequate system preparedness (*r* = −0.154), stress (*r* = −0.395), depression (*r* = −0.290), and anxiety symptoms (*r* = −0.303). The statistically significant (*p*-value all < 0.01) correlations met the pre-condition of meditation analysis.

### 3.4 Mediation effect analysis

Based on the above results and our hypotheses, we build two mediation models (Figure 1). (1) Stress and resilience as the chain mediator, perceived inadequate system preparedness as the independent factor, and depression symptoms as the dependent factor. While COVID-19 infection status and sex were considered as covariates. (2) Stress and resilience as the chain mediator, perceived inadequate system preparedness as the independent factor, and anxiety symptoms as the dependent factor. While age and marriage were considered as covariates. Model 1 and Model 2 further verified Hypotheses 1, 2, and 4 are valid, but they did not support Hypothesis 3.

**Figure 3:**
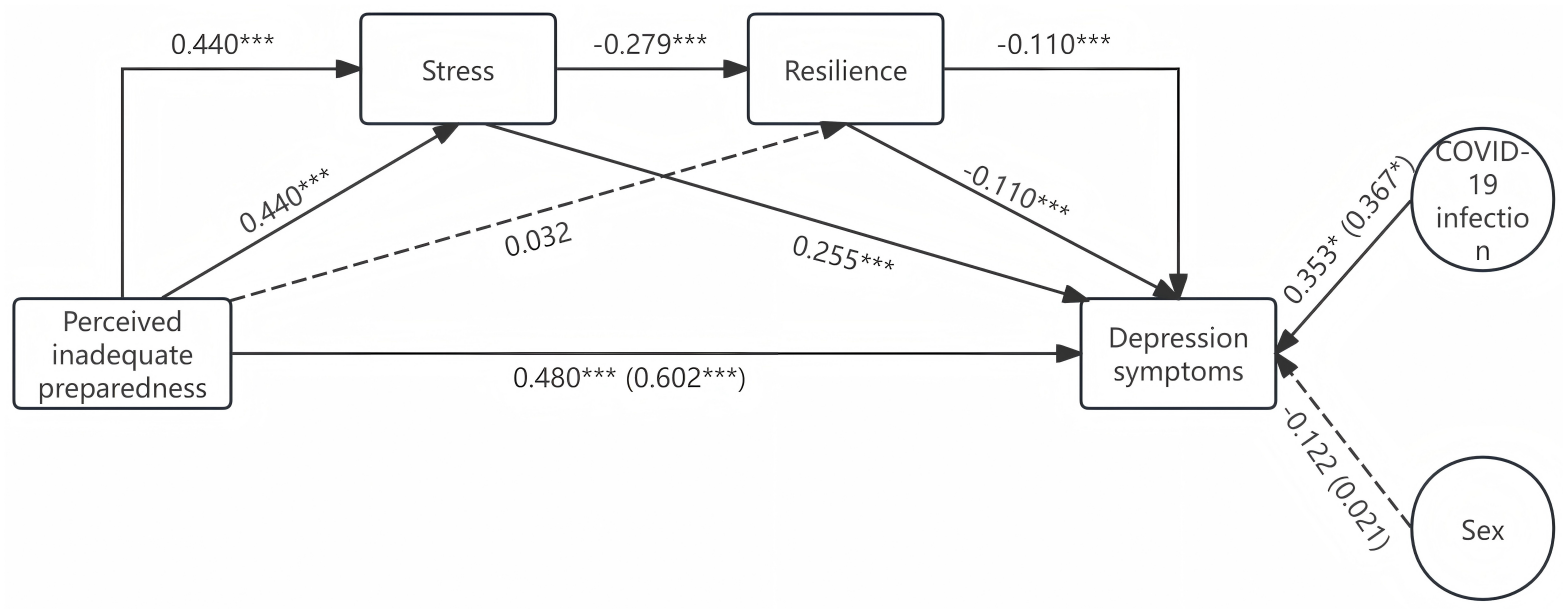
Model 1. The dotted lines indicate non-significant statistical effects, while the parentheses denote the overall effect. *p < 0.05, ***p < 0.001.

Model 1 indicated that perceived inadequate system preparedness is positively associated with depression symptoms and stress mediates the effect of perceived inadequate system preparedness on depression symptoms. The unstandardized direct predictive effect of perceived inadequate system preparedness on depression symptoms was 0.480 [bias-corrected 95 % confidence interval (0.444, 0.516)]. (The confidence interval excludes the null hypothesis value of 0, which supports Hypotheses 1) The indirect effects were as follows: in the perceived inadequate system preparedness-stress-depression symptoms pathway, the indirect effect was 0.112 [bias-corrected 95 % confidence interval (0.089, 0.138)]. (The confidence interval excludes the null hypothesis value of 0, which supports Hypotheses 2) While resilience cannot mediate the effect of perceived inadequate system preparedness on depression symptoms. In the perceived inadequate system preparedness-resilience-depression symptoms pathway, the indirect effect was −0.004 [bias-corrected 95% confidence interval (−0.009, 0.001)]. (The confidence interval encompasses the null value, which does not support Hypotheses 3) However, stress and resilience act as a chain mediator in the impact of perceived inadequate system preparedness on depression symptoms. The indirect effect of perceived inadequate system preparedness-stress-resilience-depression symptoms pathway was 0.014 [bias-corrected 95% confidence interval (0.006, 0.022)]. (The confidence interval excludes the null hypothesis value of 0, which supports Hypotheses 4) In addition, the COVID-19 infection status has significant direct [0.353, bias-corrected 95% confidence interval (0.030, 0.675)] and total [0.367, bias-corrected 95% confidence interval (0.007, 0.727)] effects on depression symptoms. However, no significant direct [−0.122, bias-corrected 95% confidence interval (−0.577, 0.334)] and total [0.021, bias-corrected 95% confidence interval (−0.488, 0.530)] effects of sex on depression symptoms. The results are shown in Table 2. The visualization of this model is shown in Figure 3.

**Table 2:** Total, direct, and indirect effects of perceived inadequate system preparedness on depression symptoms.

**Effects**	**Paths**	**Unstandardized estimates**	**Bootstrap**
			**Bias-corrected**
			**95%CI**
Direct	Perceived inadequate system preparedness → depression symptoms	0.480^***^	0.444 to 0.516
	Perceived inadequate system preparedness → stress	0.440^***^	0.386 to 0.493
	Perceived inadequate system preparedness → resilience	0.032	−0.011 to 0.075
	Stress → depression symptoms	0.255^***^	0.212 to 0.299
	Stress → resilience	−0.279^***^	−0.327 to −0.231
	Resilience → depression symptoms	−0.110^***^	−0.168 to −0.053
	Sex → depression symptoms	−0.122	−0.577 to 0.334
	COVID-19 infection status → depression symptoms	0.353^*^	0.030 to 0.675
Indirect	Perceived inadequate system preparedness → stress → depression symptoms	0.112^***^	0.089 to 0.138
	Perceived inadequate system preparedness → resilience → depression symptoms	−0.004	−0.009 to 0.001
	Perceived inadequate system preparedness → stress → resilience → depression symptoms	0.014^***^	0.006 to 0.022
Total	Perceived inadequate system preparedness → depression symptoms	0.602^***^	0.567 to 0.637
	Sex → depression symptoms	0.021	−0.488 to 0.530
	COVID-19 infection status → depression symptoms	0.367^*^	0.007 to 0.727

^*^p < 0.05, ^***^p < 0.001.

**Figure 4:**
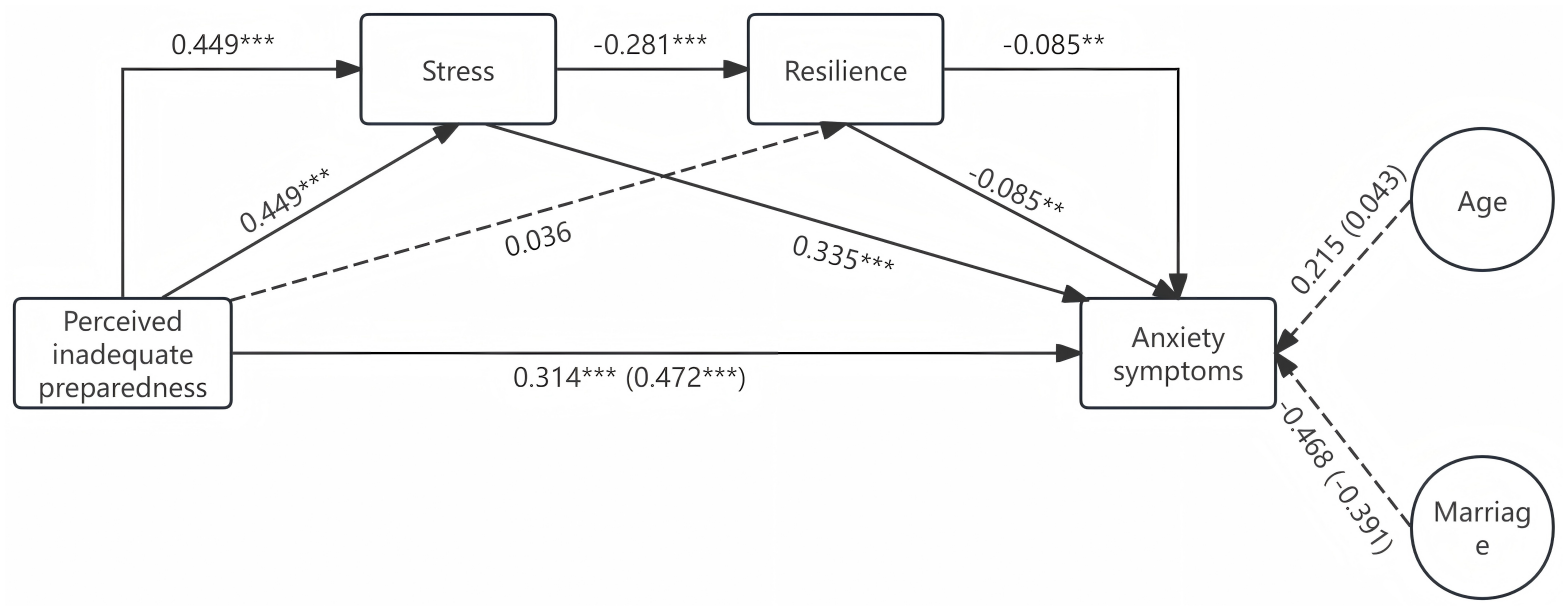
Model 2. The dotted lines indicate non-significant statistical effects, while the parentheses denote the overall effect. **p < 0.01, ***p < 0.001.

Model 2 indicated that perceived inadequate system preparedness is positively associated with anxiety symptoms and stress mediates the effect of perceived inadequate system preparedness on anxiety symptoms. The unstandardized direct predictive effect of perceived inadequate system preparedness on anxiety symptoms was 0.314 [bias-corrected 95% confidence interval (0.277, 0.351)]. (The confidence interval excludes the null hypothesis value of 0, which supports Hypotheses 1) The indirect effects were as follows: in the perceived inadequate system preparedness-stress-anxiety symptoms pathway, the indirect effect was 0.151 [bias-corrected 95% confidence interval (0.128, 0.175)]. (The confidence interval excludes the null hypothesis value of 0, which supports Hypotheses 2) While resilience cannot mediate the effect of perceived inadequate system preparedness on anxiety symptoms. In the perceived inadequate system preparedness-resilience-anxiety symptoms pathway, the indirect effect was −0.003 [bias-corrected 95% confidence interval (−0.008, 0.001)]. (The confidence interval encompasses the null value, which does not support Hypotheses 3) However, stress and resilience act as a chain mediator in the impact of perceived inadequate system preparedness on anxiety symptoms. The indirect effect of perceived inadequate system preparedness-stress-resilience-anxiety symptoms pathway was 0.011[bias-corrected 95% confidence interval (0.003, 0.019)]. (The confidence interval excludes the null hypothesis value of 0, which supports Hypotheses 4) While no significant direct [0.215, bias-corrected 95% confidence interval (−0.047, 0.477)] and total [0.043, bias-corrected 95% confidence interval (−0.263, 0.349)] effects of age on depression symptoms. Marriage did not exhibit significant direct [−0.468, bias-corrected 95% confidence interval (−1.181, 0.245)] or total effects [−0.391, bias-corrected 95% confidence interval (−1.226, 0.444)] on depression symptoms also. The results are shown in Table 3. A visualization of this model is in Figure 4.

**Table 3:** Total, direct, and indirect effects of perceived inadequate system preparedness on anxiety symptoms.

**Effects**	**Paths**	**Unstandardized estimates**	**Bootstrap**
			**Bias-corrected**
			**95%CI**
Direct	Perceived inadequate system preparedness → anxiety symptoms	0.314^***^	0.277 to 0.351
	Perceived inadequate system preparedness → stress	0.449^***^	0.395 to 0.503
	Perceived inadequate system preparedness → resilience	0.036	−0.007 to 0.080
	Stress → anxiety symptoms	0.335^***^	0.292 to 0.379
	Stress → resilience	−0.281^***^	−0.329 to −0.233
	Resilience → anxiety symptoms	−0.085^**^	−0.143 to −0.027
	Age → anxiety symptoms	0.215	−0.047 to 0.477
	Marriage → anxiety symptoms	−0.468	−1.181 to 0.245
Indirect	Perceived inadequate system preparedness → stress → anxiety symptoms	0.151^***^	0.128 to 0.175
	Perceived inadequate system preparedness → resilience → anxiety symptoms	−0.003	−0.008 to 0.001
	Perceived inadequate system preparedness → stress → resilience → anxiety symptoms	0.011^***^	0.003 to 0.019
Total	Perceived inadequate system preparedness → anxiety symptoms	0.472^***^	0.435 to 0.510
	Age → anxiety symptoms	0.043	−0.263 to 0.349
	Marriage → anxiety symptoms	−0.391	−1.226 to 0.444

^**^p < 0.01, ^***^p < 0.001.

## 4 Discussion

The results of the study show that the negative emotions of healthcare professionals were great after the government relaxed the epidemic control measures. The prevalence of depression and anxiety symptoms in our sample was 32.1 and 16.2%. Rates of depression symptoms in this population overall are similar to those at the beginning of the pandemic, although rates of anxiety symptoms remain slightly lower (59). This may be because they understand and grasp the nature of the COVID-19 pathogen itself better than they did at the beginning of the outbreak, and anxiety can gradually diminish over time (60). Our mediation analysis revealed that perceived inadequate system preparedness not only directly impacts the negative emotions of these individuals but also indirectly influences these emotions through stress and the chain mediation of stress and resilience, while it does not have an indirect impact on negative emotions solely through resilience. Furthermore, we observed a significant association between COVID-19 infection status and depression symptoms, while sex, age, and marriage did not demonstrate any impact on negative emotions. Although these variables were identified as independent predictors of negative emotions in logistic regression analysis.

### 4.1 Effects of perceived inadequate system preparedness on negative emotions

Our study suggests that perceived inadequate system preparedness can directly predict symptoms of depression and anxiety, aligning with the findings of Afulani et al. (30) to some extent. However, their investigation focused on stress and burnout as indicators and without exploring these factors within a mediating model. In fact, perceived inadequate system preparedness of specific actions is closely associated with the negative emotional experiences of healthcare professionals. As previously mentioned, insufficient mental health education and intervention (11), inadequate supply of personal protective equipment (12), and prolonged exposure to COVID-19 patients (13) were identified as specific actions. These researchers found that these particular measures were independently associated with negative emotions in multivariate regression analyses. In contrast, our study integrates these specific measures into a composite variable to examine its effect within a mediating model. It was observed that perceived inadequate system preparedness exerted a moderate direct effect on the symptoms of depression (0.480) and anxiety (0.314). The predictive effect remained significant after the inclusion of mediating variables, which supports Hypothesis 1. The greater the level of unpreparedness, the more pronounced the negative emotions experienced by healthcare professionals. This phenomenon may arise from insufficient preparation or the implementation of inadequate measures that undermine healthcare professionals' job satisfaction, elevate stress levels, and precipitate burnout, among other consequences. Research has also shown that perceived experiences and enacted negative feelings can have profound psychological impacts (61–63), underlining the importance of understanding the perceptions of system preparedness of healthcare professionals as an early indicator of potential mental health challenges. Therefore, it is imperative to be thoroughly prepared prior to the outbreak of the epidemic. As emphasized by Belfroid et al. (64), establishing an optimal work environment for healthcare professionals is imperative in effectively addressing the epidemic outbreak. It is crucial to cater to their individual requirements and ensure comprehensive support from both the team and management.

### 4.2 Stress mediates between perceived inadequate system preparedness and negative emotions

The findings suggest that perceived inadequate system preparedness may indirectly contribute to the manifestation of negative emotional symptoms by heightening the stress levels experienced by medical personnel. The mediating effects of stress on the relationship between perceived inadequate system preparedness and depression or anxiety symptoms were found to be 0.112 and 0.151, respectively, accounting for 18.6 and 32.0% of the total effect, thus providing support for Hypothesis 2. As previously mentioned, perceived inadequate system preparedness can heighten the stress levels experienced by healthcare professionals, particularly in the context of a pandemic. During the initial phase of the epidemic, it may have been attributed to limited understanding of the virus and preventive measures (65). While during the intermediate and later stages of the epidemic, with increasing knowledge about pathogens, medical personnel primarily experienced psychological stress due to excessive working hours (66) and inadequate PPE (67), etc. In our study, the symptoms of stress among healthcare professionals may also be associated with inadequate risk allowances (as indicated in the questionnaire), implying that distinct preparatory measures are required at different time stages of the epidemic. Moreover, stress experienced by healthcare professionals is significantly associated with negative emotional symptoms. Numerous researchers have demonstrated that stress can induce negative emotional symptoms through social psychological and physiological mechanisms. It is noteworthy that a singular, acute, large-scale stress event not only triggers immediate depressive and anxiety symptoms (68) but also leads to subsequent manifestation of PTSD symptoms (69). After the relaxation of the policy, a substantial influx of COVID-19 patients inundated medical facilities within a condensed timeframe, compelling hospitals to accommodate infected individuals across all departments, thereby exposing healthcare personnel to infection risks and necessitating their relentless commitment. The surge in patient numbers resulted in an acute shortage of healthcare professional and protracted working hours, collectively constituting distressing events that imposed significant psychological burdens on healthcare professionals (19). Therefore, it is imperative to adequately prepare prior to policy adjustments in order to mitigate the profound impact of the epidemic and alleviate the detrimental effects of stressful events on the mental wellbeing of healthcare professionals.

### 4.3 Resilience mediates between perceived inadequate system preparedness and negative emotions

The findings suggest that perceived inadequate system preparedness does not have an indirect impact on negative emotions through resilience as a mediating factor. The mediating effects of resilience between perceived inadequate system preparedness and depressive or anxiety symptoms were found to be non-significant (−0.004 and −0.003, respectively), thereby failing to support Hypothesis 3. This could potentially be attributed to the fact that resilience primarily acts as a protective factor against negative emotional symptoms (70) rather than directly counteracting the impact of perceived inadequate system preparedness on such emotions. Additionally, it is possible that the influence of perceived inadequate system preparedness on negative emotional symptoms is substantial, rendering the effect of resilience in countering or mitigating this impact relatively weak and not evident in the mediating model. However, a significant correlation exists between resilience and negative emotions (Direct effects on depression or anxiety symptoms were −0.110 and −0.085, respectively), which is consistent with the prevailing perspective in academia. According to Israelashvili (71), amidst the COVID-19 pandemic, enhanced resilience is associated with a greater prevalence of positive emotions, particularly among individuals experiencing heightened negative emotions. A systematic review revealed that targeted psychological interventions aimed at enhancing resilience among healthcare professionals can effectively mitigate distress and depression symptoms, albeit with a modest and transient overall impact (72). However, in this study, we contend that psychological interventions aimed at enhancing resilience following a large-scale public health emergency are comparatively less efficacious than pre-outbreak preparedness measures, as resilience appears to be insufficient in directly mitigating the adverse emotional consequences of being unprepared.

### 4.4 Role of stress and resilience in the chain mediation between perceived inadequate system preparedness and negative emotions

Results showed that stress and resilience played a chain-mediating role in the relationship between perceived inadequate system preparedness and negative emotions. The chain mediation effects of stress and resilience, although statistically small yet significant (Depression and anxiety symptoms at 0.014 and 0.011, respectively), provide empirical support for Hypothesis 4. The path effects of the model in Hypothesis 2, namely 0.112 and 0.151, respectively, are observed to decrease to 0.014 and 0.011 correspondingly after incorporating resilience as an indicator in Hypothesis 4. This finding suggests that resilience has the potential to alleviate the adverse emotional impact experienced by healthcare professionals due to stress arising from perceived inadequate system preparedness, although resilience does not directly mitigate the impact of perceived inadequate system preparedness on negative emotions in Hypothesis 3. The protective effect of resilience may be attributed to the mediating role of it in mitigating the impact of stress on negative emotions. As Chen et al. (41) found, resilience can buffer the impact of COVID-19 stress on depression symptoms. Yildirim et al. (73) discovered that resilience can alleviate the impact of coronavirus fear on depression and anxiety, while also establishing a close association between coronavirus fear and stress. In fact, resilience is defined as an individual's capacity to maintain or restore a state of relative stability in their mental and physical functioning amidst challenging life events and adversity (74). Pandemics such as COVID-19 exemplify formidable challenges or adversities in the realm of public health (75). The greater one's resilience, the stronger their capacity to withstand challenging events (76). Consequently, psychosocial interventions aimed at bolstering resilience can also hold certain significant implications for safeguarding the mental wellbeing of medical personnel.

### 4.5 The effects of other variables on negative emotions

We have also identified additional factors that exhibit a strong correlation with symptoms of depression or anxiety. In the stepwise regression logistic model, we observed that COVID-19 infection status and sex were independent predictors of depression symptoms, while age and marriage were independent predictors of anxiety symptoms. After incorporating these variables into the final mediator model, only the COVID-19 infection remained statistically significant, consistent with findings from prior studies. As Ergai et al. (77) discovered a positive correlation between healthcare workers' concerns regarding personal infection and elevated levels of depressive symptoms, while Deguchi et al. (78) identified a higher risk of depressive symptoms associated with a diagnosis or suspected infection of COVID-19. It is therefore imperative to safeguard the mental wellbeing of healthcare professionals who have contracted COVID-19. However, there is limited evidence to suggest that sex, age, and marital status exert a direct or overall significant influence on depression or anxiety symptoms. These findings are inconsistent with the results reported in certain studies (79–81). It could be attributed to two factors. Firstly, instead of employing mediation models, single-variable and multiple-variable regression methods were utilized of them. Secondly, our model encompassed perceived inadequate system preparedness, stress levels, etc., and during the peak of the epidemic following policy relaxation, nearly all healthcare professionals irrespective of sex, age or marital status were engaged in high-intensity work with comparable pressure. Consequently, the impact of these variables on negative emotions remained inconspicuous. However, it is noteworthy that directing attention toward young or aged over 50 and unmarried or divorced female healthcare professionals may hold certain significant implications for safeguarding their mental wellbeing, given the persistent significance of these variables in prior studies as well as our multivariate analysis.

In summary, we have substantiated the validity of Hypotheses 1, 2, and 4 through rigorous univariate and multivariate analyses, correlation analysis, as well as a comprehensive mediating model. Furthermore, Hypothesis 3 has been refuted conclusively. Moreover, our investigation has unveiled additional variables associated with negative emotions such as COVID-19 infection. To the best of our knowledge, this study represents the first investigation into the pathway of perceived inadequate system preparedness impact on negative emotions experienced by healthcare professional during the post-epidemic control period in China. In fact, several countries had already implemented open policies or pursued herd immunity before China relaxed its measures (82). However, their transition from stringent control measures to open policies appeared inadequately prepared, as evidenced by the rapid surge in confirmed cases and fatalities upon reopening of many cities in countries like the United States and Australia (83). A study demonstrates that without lockdowns, public health interventions, economic measures, and mask usage, the incidence rate of COVID-19 would have significantly increased (84). Moreover, these measures exhibit greater effectiveness when implemented earlier. Therefore, adequate system preparedness and related perceptions not only safeguards mental wellbeing but also mitigates the incidence and mortality rates, thereby minimizing the impact of public health events on society.

## 5 Limitations

The limitations of this study are likely to be as follows. Firstly, it was a non-probability sample and cross-sectional study. Therefore, the power to prove causality is weak. Secondly, the multifaceted nature of inadequate preparedness should be acknowledged. It is important to recognize that our measured perceived inadequate system preparedness may not encompass the entirety of the phenomenon. While our study focuses on healthcare professionals' perceptions of system preparedness rather than comprehensive system-level assessment, these perceptions provide crucial insights into how preparedness measures translate into frontline experiences. Future research could integrate these findings with organizational-level metrics to provide a more complete picture of system preparedness. Thirdly, questionnaire measurements cannot substitute structured interviews. The scores obtained from the questionnaires may have a certain degree of subjectivity. Therefore, future research should prioritize conducting longitudinal, randomized studies that delve deeper into the concept of perceived inadequate system preparedness in order to provide viable strategies or policy recommendations for managing large-scale public health emergencies.

## 6 Conclusions

Our study validates that inadequate preparation is a direct predictor of negative emotions and can indirectly predict negative emotions through the mediating role of stress, as well as the chain mediation of stress and resilience. However, it does not exert an indirect effect on negative emotions through resilience as a mediator. Additionally, contracting COVID-19 also serves as a predictor for depression symptoms. Therefore, in order to safeguard the mental wellbeing of healthcare professionals and effectively address future public health crises, it is most imperative to proactively prepare and respond prior to and during outbreaks. This includes measures such as optimizing work schedules to mitigate excessive hours and allowing adequate rest for healthcare professionals; ensuring an ample supply of masks, medications, etc.; bolstering workforce capacity and surge capabilities; as well as providing psychological counseling and intervention for them (as indicated by the “Perceived Inadequate System Preparedness” questionnaire). Additionally, it is crucial to implement psychosocial interventions with the objective of enhancing resilience and concerning the healthcare professionals who have already contracted COVID-19. Furthermore, directing attention toward young individuals, those over 50 years old, unmarried or divorced individuals, and female healthcare workers holds potential for significant benefits.

## Data Availability

The raw data supporting the conclusions of this article will be made available by the authors, without undue reservation.
