# Supplementary material for: Healthcare professionals' perceptions of system preparedness during public health emergencies: a path analysis of mental health impacts
Source: Front Public Health. 2025 Apr 17;13:1449207. doi: 10.3389/fpubh.2025.1449207 (PMC12043465; doi:10.3389/fpubh.2025.1449207)
Supplement: Supplementary file 1 [file Supplementary_file_1.docx]

**(English Version) Mental health questionnaire for medical staff (selected questions)**

**Hello, to those who participated in the questionnaire survey! This questionnaire attempts to study the current mental health status of medical staff, and tries to call for possible measures (such as strengthening psychological intervention and counseling, improving the distribution of materials and drugs, timely rest, reasonably improving the security of medical staff and medical students, etc.). This questionnaire will not disclose any of your personal privacy and is only used for the collection and analysis of psychological information. It may take a few minutes to fill in the questionnaire. If you feel uncomfortable with any of the questions, you could withdraw at any time and the questionnaire will become invalid.**

1. Your gender: [Multiple choice] *

| ○ Male | ○Female |  |  |  |  |  |  |
| --- | --- | --- | --- | --- | --- | --- | --- |

2. Your age group: [Multiple choice] *

| ○ <18 | ○18~25 | ○26~30 | ○31~40 |
| --- | --- | --- | --- |
| ○41~50 | ○51~60 | ○ >60 |  |

3. Please choose provinces, cities and regions: [Fill in the blanks] *

_________________________________

4. Your identity:

[Multiple choice] *

| ○ Graduates, interns, and residents  ○ Doctors  ○ Nurses  ○ Others(administrative roles, auxiliary personnel, pharmaceutical staff, etc.) |
| --- |

5. Your working life: [Multiple choice] *

| ○ <2 years |
| --- |
| ○ 2~5 years |
| ○ 5~ 10 years |
| ○> 10 years |

6. Your degree: [Multiple choice] *

| ○ Below undergraduate level |
| --- |
| ○ Undergraduate |
| ○ Master |
| ○ Doctor and above |

8. Your current COVID -19 infection status: [Multiple choice] *

| ○ Uninfected |
| --- |
| ○ Infected with obvious symptoms |
| ○Almost or fully recovered |

9. Your marital status [Multiple choice] *

| ○ Unmarried |
| --- |
| ○ Married |
| ○ Divorced |

11. Your monthly income: [Multiple choice] *

| ○<6000 yuan |
| --- |
| ○ 6000-10000 yuan |
| ○ 10000-20000 yuan |
| ○>20000 yuan |

Thank you for filling in here. The following are some psychological assessments. Please answer whether there are the conditions described below and their frequency according to your situation in the past two weeks.

12. Little interest or pleasure in doing things [Multiple choice]*

| ○A. Not at all |
| --- |
| ○B. Several days |
| ○C. More than half the days |
| ○D. Nearly every day |

13. Trouble falling or staying asleep, or sleeping too much [Multiple choice] *

| ○A. Not at all |
| --- |
| ○B. Several days |
| ○C. More than half the days |
| ○D. Nearly every day |

14. Feeling down, depressed, or hopeless [Multiple choice] *

| ○A. Not at all |
| --- |
| ○B. Several days |
| ○C. More than half the days |
| ○D. Nearly every day |

15.Feeling tired or having little energy [Multiple choice] *

| ○A. Not at all |
| --- |
| ○B. Several days |
| ○C. More than half the days |
| ○D. Nearly every day |

16. Poor appetite or overeating [Multiple choice] *

| ○A. Not at all |
| --- |

| ○B. Several days |
| --- |
| ○C. More than half the days |
| ○D. Nearly every day |

17.Feeling bad about yourself — or that you are a failure or have let yourself or your family down [Multiple choice] *

| ○A. Not at all |
| --- |
| ○B. Several days |
| ○C. More than half the days |
| ○D. Nearly every day |

18.Trouble concentrating on things, such as reading the newspaper or watching television [Multiple choice] *

| ○A. Not at all |
| --- |
| ○B. Several days |
| ○C. More than half the days |
| ○D. Nearly every day |

19.Moving or speaking so slowly that other people could have noticed? Or the opposite — being so fidgety or restless that you have been moving around a lot more than usual [Multiple choice] *

| ○A. Not at all |
| --- |
| ○B. Several days |
| ○C. More than half the days |
| ○D. Nearly everyday |

20. Thoughts that you would be better off dead or of hurting yourself in some way [Multiple choice] *

| ○A. Not at all |
| --- |

| ○B. Several days |
| --- |
| ○C. More than half the days |
| ○D. Nearly every day |

21. Feeling nervous, anxious or on edge [Multiple choice] *

| ○A. Not at all |
| --- |
| ○B. Several days |
| ○C. More than half the days |
| ○D. Nearly every day |

22. Not being able to stop or control worrying [Multiple choice] *

| ○A. Not at all |
| --- |
| ○B. Several days |
| ○C. More than half the days |
| ○D. Nearly every day |

23. Worrying too much about different things [Multiple choice] *

| ○A. Not at all |
| --- |
| ○B. Several days |
| ○C. More than half the days |
| ○D. Nearly every day |

24. Trouble relaxing [Multiple choice] *

| ○A. Not at all |
| --- |
| ○B. Several days |

| ○C. More than half the days |
| --- |
| ○D. Nearly every day |

25. Being so restless that it is hard to sit still [Multiple choice] *

| ○A. Not at all |
| --- |
| ○B. Several days |
| ○C. More than half the days |
| ○D. Nearly every day |

26. Becoming easily annoyed or irritable [Multiple choice] *

| ○A. Not at all |
| --- |
| ○B. Several days |
| ○C. More than half the days |
| ○D. Nearly every day |

27. Feeling afraid as if something awful might happen [Multiple choice] *

| ○A. Not at all |
| --- |
| ○B. Several days |
| ○C. More than half the days |
| ○D. Nearly every day |

Please recall your situation for the last month and choose the option that best suits you

28. In the last month, how often have you been upset because of something that happened unexpectedly? [Multiple choice]

| ○ Never |
| --- |
| ○ Almost never |

| ○ Sometimes |
| --- |
| ○ Fairly often |
| ○ Very often |

29. How often in the past month did you feel unable to control the important things in your life? [Multiple choice]

*

| ○ Never |
| --- |
| ○ Almost never |
| ○ Sometimes |
| ○ Fairly often |
| ○ Very often |

30. In the last month, how often have you felt nervous and "stressed"? [Multiple choice] *

| ○ Never |
| --- |
| ○ Almost never |
| ○ Sometimes |
| ○ Fairly often |
| ○ Very often |

31. How much time in the last month have you felt confident about your ability to deal with your personal problems? [Multiple choice] *

| ○ Never |
| --- |
| ○ Almost never |
| ○ Sometimes |
| ○ Fairly often |

| ○ Very often |
| --- |

32. How often in the last month did you feel things going as you expected? [Multiple choice] *

| ○ Never |
| --- |
| ○ Almost never |
| ○ Sometimes |
| ○ Fairly often |
| ○ Very often |

33. In the last month, how often have you found that you could not cope with all the things that you have to do? [Multiple choice] *

| ○ Never |
| --- |
| ○ Almost never |
| ○ Sometimes |
| ○ Fairly often |
| ○ Very often |

34. In the last month, how often have you been able to control irritations in your life? [Multiple choice] *

| ○ Never |
| --- |
| ○ Almost never |
| ○ Sometimes |
| ○ Fairly often |
| ○ Very often |

35. How often in the last month did you feel confident in dealing with things (all in your control)? [Multiple choice] *

| ○ Never |
| --- |
| ○ Almost never |
| ○ Sometimes |
| ○ Fairly often |
| ○ Very often |

36. In the last month, how often have you been angered because of things that happened that were outside of your control? [Multiple choice] *

| ○ Never |
| --- |
| ○ Almost never |
| ○ Sometimes |
| ○ Fairly often |
| ○ Very often |

37. In the last month, how often have you felt difficulties were piling up so high that you could not overcome them? [Multiple choice] *

| ○ Never |
| --- |
| ○ Almost never |
| ○ Sometimes |
| ○ Fairly often |
| ○ Very often |

38. Which of the following do you think is the main cause of your possible bad mood and rate it (The higher the score, the more you worried and more in line with the current situation) [Matrix scale Question]

*

|  | 0 | 1 | 2 | 3 | 4 | 5 |
| --- | --- | --- | --- | --- | --- | --- |
| ① Lack of psychological counseling measures | ○ | ○ | ○ | ○ | ○ | ○ |
| ②Lack of masks, medicine and other supplies | ○ | ○ | ○ | ○ | ○ | ○ |
| ③Salaries need to be improved during the epidemic | ○ | ○ | ○ | ○ | ○ | ○ |
| ④An excess of infected patients |  |  |  |  |  |  |
| ⑤Working long hours with no breaks |  |  |  |  |  |  |

39. Please rate the following items on a scale of 1-5 from lowest to highest, where 1 means it does not describe you at all and 5 means it describes you very well [Matrix Scale Question] *

|  | 1 | 2 | 3 | 4 | 5 |
| --- | --- | --- | --- | --- | --- |
| ①I look for creative ways to  alter diffificult situations. | ○ | ○ | ○ | ○ | ○ |
| ②Regardless of what happens  to me, I believe I can control  my reaction to it. | ○ | ○ | ○ | ○ | ○ |
| ③I believe I can grow in positive ways by dealing with  difficult situations. | ○ | ○ | ○ | ○ | ○ |
| ④I actively look for ways to  replace the losses I encounter in life. | ○ | ○ | ○ | ○ | ○ |

**（Chinese Version）医务人员及一般人群心理健康调查问卷 (已选问卷问题)**

参与问卷调查的小伙伴你们好！本调查问卷试图研究一线医务人员和一般人群目前的心理健康状况，并试图对可能的措施(如加强心理干预与疏导，提高物资和药品的分配，及时休息，合理提高对医务人员和医学生的保障等）进行呼吁。本问卷不会泄露您的任何个人隐私，只作为收集和分析心理信息使用。填写问卷可能需要您几分钟的时间，如您对某些问题感到不适可以随时退出，问卷作废。填写完问卷之后会有一定数额的红包，如果您符合此次调查的对象，并同意填写的话，请您认真填写，我们会对认真填写的小伙伴发放红包，感谢您的参与！

1. 您的性别： [单选题] *

| ○男 | ○女 | ○其他 |  |  |  |  |  |
| --- | --- | --- | --- | --- | --- | --- | --- |

2. 您的年龄段： [单选题] *

| ○18岁以下 | ○18~25 | ○26~30 | ○31~40 |
| --- | --- | --- | --- |
| ○41~50 | ○51~60 | ○60以上 |  |

3. 请选择省份城市与地区: [填空题] *

_________________________________

4. 您的身份： [单选题] *

| ○研究生、实习生、规培生等 |
| --- |
| ○医生 |
| ○护士 |
| ○其他（药剂师、后勤人员、医院行政岗位等） |

5. 您的工作年限： [单选题] *

| ○<2年 |
| --- |
| ○2~5年 |
| ○5~10年 |
| ○>10年 |

6. 您已取得的学历： [单选题] *

| ○本科以下 |
| --- |
| ○本科 |
| ○硕士 |
| ○博士及以上 |

8. 您目前的新冠感染状态： [单选题] *

| ○未感染 |
| --- |
| ○已感染，有症状 |
| ○快康复或者完全康复 |

9. 您的婚姻状况 [单选题] *

| ○未婚 |
| --- |
| ○已婚 |
| ○离异 |

11. 您的月收入： [单选题] *

| ○<6000元 |
| --- |
| ○6000-10000元 |
| ○10000-20000元 |
| ○>20000元 |

感谢您已经填到这里，以下是一些心理测评，请您根据过去两周的情况回答是否存在下列描述的状况及频率，请看清楚问题后选择符合您的选项，根据直觉作答即可

12. 做事时提不起劲或没有兴趣 [单选题] *

| ○A.完全不会 |
| --- |
| ○B.好几天 |
| ○C.一半以上天数 |
| ○D.几乎每天 |

13. 入睡困难、睡不安稳或睡眠过多 [单选题] *

| ○A.完全不会 |
| --- |
| ○B.好几天 |
| ○C.一半以上天数 |
| ○D.几乎每天 |

14. 感到心情低落、沮丧或绝望 [单选题] *

| ○A.完全不会 |
| --- |
| ○B.好几天 |
| ○C.一半以上天数 |
| ○D.几乎每天 |

15. 感觉疲倦或没有活力 [单选题] *

| ○A.完全不会 |
| --- |
| ○B.好几天 |
| ○C.一半以上天数 |
| ○D.几乎每天 |

16. 食欲不振或吃太多 [单选题] *

| ○A.完全不会 |
| --- |
| ○B.好几天 |
| ○C.一半以上天数 |
| ○D.几乎每天 |

17. 觉得自己很糟或觉得自己很失败，或让自己或家人失望 [单选题] *

| ○A.完全不会 |
| --- |
| ○B.好几天 |
| ○C.一半以上天数 |
| ○D.几乎每天 |

18. 对事物专注有困难，例如:阅读报纸或看电视时 [单选题] *

| ○A.完全不会 |
| --- |
| ○B.好几天 |
| ○C.一半以上天数 |
| ○D.几乎每天 |

19. 动作或说话速度缓慢到别人已经察觉?或正好相反，烦躁或坐立不安、动来动去的情况更胜于平常 [单选题] *

| ○A.完全不会 |
| --- |
| ○B.好几天 |
| ○C.一半以上天数 |
| ○D.几乎每天 |

20. 有不如死掉或用某种方式伤害自己的念头 [单选题] *

| ○A.完全不会 |
| --- |
| ○B.好几天 |
| ○C.一半以上天数 |
| ○D.几乎每天 |

21. 感觉紧张、焦虑或急切 [单选题] *

| ○A.完全不会 |
| --- |
| ○B.好几天 |
| ○C.一半以上天数 |
| ○D.几乎每天 |

22. 不能够停止或控制担忧 [单选题] *

| ○A.完全不会 |
| --- |
| ○B.好几天 |
| ○C.一半以上天数 |
| ○D.几乎每天 |

23. 对各种各样的事情担忧过多 [单选题] *

| ○A.完全不会 |
| --- |
| ○B.好几天 |
| ○C.一半以上天数 |
| ○D.几乎每天 |

24. 很难放松下来 [单选题] *

| ○A.完全不会 |
| --- |
| ○B.好几天 |
| ○C.一半以上天数 |
| ○D.几乎每天 |

25. 由于不安而无法静坐 [单选题] *

| ○A.完全不会 |
| --- |
| ○B.好几天 |
| ○C.一半以上天数 |
| ○D.几乎每天 |

26. 变得容易烦恼或急躁 [单选题] *

| ○A.完全不会 |
| --- |
| ○B.好几天 |
| ○C.一半以上天数 |
| ○D.几乎每天 |

27. 感到似乎将有可怕的事情发生而害怕 [单选题] *

| ○A.完全不会 |
| --- |
| ○B.好几天 |
| ○C.一半以上天数 |
| ○D.几乎每天 |

28. 在过去的一个月里，你有多少时间因为发生意外的事情而感到心烦意乱？ [单选题] *

| ○从未有 |
| --- |
| ○几乎没有 |
| ○偶尔 |
| ○经常 |
| ○非常多 |

29. 在过去的一个月里，有多少时间你感到无法掌控生活中重要的事情？ [单选题] *

| ○从未有 |
| --- |
| ○几乎没有 |
| ○偶尔 |
| ○经常 |
| ○非常多 |

30. 在过去的一个月里，有多少时间你感觉到神经紧张或“快被压垮了”？ [单选题]*

| ○从未有 |
| --- |
| ○几乎没有 |
| ○偶尔 |
| ○经常 |
| ○非常多 |

31. 在过去的一个月里，有多少时间你对自己处理个人问题的能力感到有信心？ [单选题] *

| ○从未有 |
| --- |
| ○几乎没有 |
| ○偶尔 |
| ○经常 |
| ○非常多 |

32. 在过去的一个月里，有多少时间你感到事情发展和你预料的一样？ [单选题] *

| ○从未有 |
| --- |
| ○几乎没有 |
| ○偶尔 |
| ○经常 |
| ○非常多 |

33. 在过去的一个月里，有多少时间你发现自己无法应付那些你必须去做的事情。 [单选题] *

| ○从未有 |
| --- |
| ○几乎没有 |
| ○偶尔 |
| ○经常 |
| ○非常多 |

34. 在过去的一个月里，日常生活中有多少时间你能够控制自己的愤怒情绪？ [单选题] *

| ○从未有 |
| --- |
| ○几乎没有 |
| ○偶尔 |
| ○经常 |
| ○非常多 |

35. 在过去的一个月里，有多少时间你感到处理事情得心应手(事情都在你的控制之中)？ [单选题] *

| ○从未有 |
| --- |
| ○几乎没有 |
| ○偶尔 |
| ○经常 |
| ○非常多 |

36. 在过去的一个月里，有多少时间你因为一些超出自己控制能力的事情而感到愤怒？ [单选题] *

| ○从未有 |
| --- |
| ○几乎没有 |
| ○偶尔 |
| ○经常 |
| ○非常多 |

37. 在过去的一个月里，有多少时间你感到问题堆积如山，已经无法逾越？ [单选题] *

| ○从未有 |
| --- |
| ○几乎没有 |
| ○偶尔 |
| ○经常 |
| ○非常多 |

38. 您认为您可能的不良情绪主要来自于以下哪件事，并予以打分（分数越高说明该项越影响您的情绪）[矩阵量表题] *

|  | 0 | 1 | 2 | 3 | 4 | 5 |
| --- | --- | --- | --- | --- | --- | --- |
| ①心理疏导和干预措施缺乏 | ○ | ○ | ○ | ○ | ○ | ○ |
| ②缺少口罩、药品等保障资源 | ○ | ○ | ○ | ○ | ○ | ○ |
| ③目前薪金待遇有待提高 | ○ | ○ | ○ | ○ | ○ | ○ |
| ④阳性病人过多 | ○ | ○ | ○ | ○ | ○ | ○ |
| ⑤工作时间过长，没有休息时间 | ○ | ○ | ○ | ○ | ○ | ○ |

39. 请对以下项目进行打分（1-5分代表由低到高），其中1表示它根本没有描述你，5表示它很好地描述了你[矩阵量表题] *

|  | 1 | 2 | 3 | 4 | 5 |
| --- | --- | --- | --- | --- | --- |
| ①在困难的处境中，我会寻求创造性的方法去改变或逆转 | ○ | ○ | ○ | ○ | ○ |
| ②无论我遇到什么，我相信自己可以控制自己的情绪反应 | ○ | ○ | ○ | ○ | ○ |
| ③我相信，我可以以积极的方式处理困难的情况，并加以成长 | ○ | ○ | ○ | ○ | ○ |
| ④我会积极的寻找方法来弥补生活中的遗憾或者看淡失败 | ○ | ○ | ○ | ○ | ○ |
