# Supplementary material for: Healthcare professionals' perceptions of system preparedness during public health emergencies: a path analysis of mental health impacts
Source: Front Public Health. 2025 Apr 17;13:1449207. doi: 10.3389/fpubh.2025.1449207 (PMC12043465; doi:10.3389/fpubh.2025.1449207)
Supplement: Supplementary file 2 [file Supplementary_file_2.docx]

**Table S1** Reliability analysis of Perceived inadequate system preparedness

Total Cronbach’s alpha

|  | Cronbach’s alpha based on standardized items | Item |
| --- | --- | --- |
| Total | 0.916 | 5 |

The Cronbach's alpha for each item (if the item is removed)

| Item | Cronbach's alpha (if the item deleted) |
| --- | --- |
| Lack of psychological counseling measures | 0.902 |
| Lack of masks, medicine and other supplies | 0.906 |
| Salaries need to be improved during the epidemic | 0.882 |
| An excess of infected patients | 0.877 |
| Working long hours with no breaks | 0.914 |

**Table S2** Exploratory factor analysis of Perceived inadequate system preparedness

Kaiser–Meyer–Olkin and Bartlett’s test

|  | Value |
| --- | --- |
| KMO | 0.886 |
| Bartlett’s test | χ2=3038.803, p<0.001 |

Communalities

| Item | Communalities |
| --- | --- |
| Lack of psychological counseling measures | 0.719 |
| Lack of masks, medicine and other supplies | 0.696 |
| Salaries need to be improved during the epidemic | 0.838 |
| An excess of infected patients | 0.869 |
| Working long hours with no breaks | 0.636 |

Principal component factor analysis

| Component | Eigenvalue | | |
| --- | --- | --- | --- |
|  | Total | Individual % | Accumulation % |
| 1 | 3.759 | 75.171 | 75.171 |
| 2 | 0.482 | 9.649 | 84.820 |
| 3 | 0.360 | 7.197 | 92.017 |
| 4 | 0.241 | 4.824 | 96.841 |
| 5 | 0.158 | 3.159 | 100.000 |

Factor loadings

| Item | Factor loadings |
| --- | --- |
| Lack of psychological counseling measures | 0.848 |
| Lack of masks, medicine and other supplies | 0.834 |
| Salaries need to be improved during the epidemic | 0.916 |
| An excess of infected patients | 0.932 |
| Working long hours with no breaks | 0.798 |

**Table S3**

Factors associated with depression symptoms (N=826)

|  |  |  | **Univariate analyses** | |  | **Logistic regression analysis^#^** | |
| --- | --- | --- | --- | --- | --- | --- | --- |
| **Independent variables, n (%)** | **No-minor depression** | **Moderate-severe depression** | **χ2** | **p-value** |  | **Adjusted OR (95%CI)** | **p-value** |
|  | **561 (67.9)** | **265 (32.1)** |  |  |  |  |  |
| **Gender** |  |  |  |  |  |  |  |
| **Male** | **202 (36)** | **76 (28.7)** |  |  |  | **—** | **—** |
| **Female** | **359 (64)** | **189 (71.3)** | **4.33** | **0.037** |  | **1.48 (0.99,2.21)** | **0.053** |
| **Age, years** |  |  |  |  |  |  |  |
| **18~25** | **236 (42.1)** | **88 (33.2)** |  |  |  |  |  |
| **26~30** | **89 (15.9)** | **44 (16.6)** |  |  |  |  |  |
| **31~40** | **113 (20.1)** | **79 (29.8)** |  |  |  |  |  |
| **41~50** | **63 (11.2)** | **35 (13.2)** |  |  |  |  |  |
| **>50** | **60 (10.7)** | **19 (7.2)** | **2.71** | **0.1** |  |  |  |
| **Type of work** |  |  |  |  |  |  |  |
| **Fellows in training** | **219(39.0)** | **47 (29.4)** |  |  |  |  |  |
| **Doctors** | **181 (32.3)** | **120 (45.3)** |  |  |  |  |  |
| **Nursers** | **93 (16.6)** | **40 (15.1)** |  |  |  |  |  |
| **Others^a^** | **68 (12.1)** | **27 (10.2)** | **13.82** | **0.003** |  |  |  |
| **Work experience, years** |  |  |  |  |  |  |  |
| **<2** | **233 (41.5)** | **94 (35.5)** |  |  |  |  |  |
| **2-5** | **74 (13.2)** | **32 (12.1)** |  |  |  |  |  |
| **5-10** | **52 (9.3)** | **40 (15.1)** |  |  |  |  |  |
| **>10** | **202 (36)** | **99 (37.4)** | **1.87** | **0.172** |  |  |  |
| **Education** |  |  |  |  |  |  |  |
| **Junior college** | **155 (27.6)** | **58 (21.9)** |  |  |  |  |  |
| **College** | **334 (59.5)** | **157 (59.2)** |  |  |  |  |  |
| **Master and above** | **72 (12.8)** | **50 (18.9)** | **6.16** | **0.013** |  |  |  |
| **COVID-19 infection status** |  |  |  |  |  |  |  |
| **Uninfected** | **69 (12.3)** | **11 (4.2)** |  |  |  | **—** | **—** |
| **Infected with obvious symptoms** | **151 (26.9)** | **114 (43)** |  |  |  | **4.61 (1.95,10.91)** | **< 0.001** |
| **Almost or fully recovered** | **341 (60.8)** | **140 (52.8)** | **28.84** | **< 0.001** |  | **3.62 (1.56,8.41)** | **0.003** |
| **Marriage** |  |  |  |  |  |  |  |
| **Single/Others** | **292 (52)** | **113 (42.6)** |  |  |  |  |  |
| **Married** | **269 (48)** | **152 (57.4)** | **6.38** | **0.012** |  |  |  |
| **Monthly income, yuan** |  |  |  |  |  |  |  |
| **<6000** | **386 (68.8)** | **166 (62.6)** |  |  |  |  |  |
| **6000-10000** | **141 (25.1)** | **76 (28.7)** |  |  |  |  |  |
| **>10000** | **34 (6.1)** | **23 (8.7)** | **3.45** | **0.063** |  |  |  |

**Note:**

^a^ Members of the support staff, including personnel in administrative, medical, and pharmacy departments, among others

**^#^** Final logistic stepwise regression model

**Table S4**

Factors associated with anxiety symptoms (N=826)

|  |  |  | **Univariate analyses** | |  | **Logistic regression analysis^#^** | |
| --- | --- | --- | --- | --- | --- | --- | --- |
| **Independent variables, n (%)** | **No-minor anxiety** | **Moderate-severe anxiety** | **χ2** | **p-value** |  | **Adjusted OR (95%CI)** | **p-value** |
|  | **692 (83.8)** | **134 (16.2)** |  |  |  |  |  |
| **Gender** |  |  |  |  |  |  |  |
| **Male** | **234 (33.8)** | **44 (32.8)** |  |  |  |  |  |
| **Female** | **458 (66.2)** | **90 (67.2)** | **0.05** | **0.826** |  |  |  |
| **Age, years** |  |  |  |  |  |  |  |
| **18~25** | **289 (41.8)** | **35 (26.1)** |  |  |  | **—** | **—** |
| **26~30** | **106 (15.3)** | **27 (20.1)** |  |  |  | **3.19 (1.41,7.24)** | **0.005** |
| **31~40** | **147 (21.2)** | **45 (33.6)** |  |  |  | **6 (2.1,17.12)** | **< 0.001** |
| **41~50** | **81 (11.7)** | **17 (12.7)** |  |  |  | **4.98 (1.55,15.98)** | **0.007** |
| **>50** | **69 (10)** | **10 (7.5)** | **5.12** | **0.024** |  | **6.23 (1.69,22.92)** | **0.006** |
| **Type of work** |  |  |  |  |  |  |  |
| **Fellows in training** | **266 (38.4)** | **31 (23.1)** |  |  |  |  |  |
| **Doctors** | **239 (34.5)** | **62 (46.3)** |  |  |  |  |  |
| **Nursers** | **109 (15.8)** | **24 (17.9)** |  |  |  |  |  |
| **Others^a^** | **78 (11.3)** | **17 (12.7)** | **12.07** | **0.007** |  |  |  |
| **Work experience, years** |  |  |  |  |  |  |  |
| **<2** | **288 (41.6)** | **39 (29.1)** |  |  |  |  |  |
| **2-5** | **84 (12.1)** | **22 (16.4)** |  |  |  |  |  |
| **5-10** | **73 (10.5)** | **19 (14.2)** |  |  |  |  |  |
| **>10** | **247 (35.7)** | **54 (40.3)** | **4.37** | **0.037** |  |  |  |
| **Education** |  |  |  |  |  |  |  |
| **Junior college** | **185 (26.7)** | **28 (20.9)** |  |  |  |  |  |
| **College** | **412 (59.5)** | **79 (59)** |  |  |  |  |  |
| **Master and above** | **95 (13.7)** | **27 (20.1)** | **4.15** | **0.042** |  |  |  |
| **COVID-19 infection status** |  |  |  |  |  |  |  |
| **Uninfected** | **71 (10.3)** | **9 (6.7)** |  |  |  |  |  |
| **Infected with symptoms** | **200 (28.9)** | **65 (48.5)** |  |  |  |  |  |
| **Almost or fully recovered** | **421 (60.8)** | **60 (44.8)** | **19.88** | **< 0.001** |  |  |  |
| **Marriage** |  |  |  |  |  |  |  |
| **Single/Others** | **350 (50.6)** | **55 (41)** |  |  |  | **—** | **—** |
| **Married** | **342 (49.4)** | **79 (59)** | **4.08** | **0.043** |  | **0.35 (0.14,0.87)** | **0.024** |
| **Monthly income, yuan** |  |  |  |  |  |  |  |
| **<6000** | **470 (67.9)** | **82 (61.2)** |  |  |  |  |  |
| **6000-10000** | **182 (26.3)** | **35 (26.1)** |  |  |  |  |  |
| **>10000** | **40 (5.8)** | **17 (12.7)** | **3.6** | **0.058** |  |  |  |

**Note:**

^a^ Members of the support staff, including personnel in administrative, medical, and pharmacy departments, among others**^#^** Final logistic stepwise regression model

**Table S5**

Means, standard deviations (SD) and correlational analyses

|  | M | SD | Perceived inadequate system preparedness | PSS-10 | BRCS | PHQ-9 | GAD-7 |
| --- | --- | --- | --- | --- | --- | --- | --- |
| Perceived inadequate system preparedness | 5.09 | 6.86 | 1 |  |  |  |  |
|  |  |  |  |  |  |  |  |
| PSS-10 | 16.79 | 6.13 | 0.494** | 1 |  |  |  |
|  |  |  |  |  |  |  |  |
| BRCS | 13.28 | 4.05 | -0.154** | -0.395** | 1 |  |  |
|  |  |  |  |  |  |  |  |
| PHQ-9 | 7.79 | 5.42 | 0.761** | 0.620** | -0.290** | 1 |  |
|  |  |  |  |  |  |  |  |
| GAD-7 | 5.22 | 4.89 | 0.657** | 0.662** | -0.303** | 0.803** | 1 |

**Note:** PSS-10: Perceived stress scale-10; BRCS: Brief Resilient Coping Scale; PHQ-9: Patient health questionnaire-9; GAD-7: Generalized anxiety disorder 7-item; M: Mean value; SD: Standard deviation.

**p<0.01.
